# Assessing the human immune system through blood transcriptomics

**DOI:** 10.1186/1741-7007-8-84

**Published:** 2010-07-01

**Authors:** Damien Chaussabel, Virginia Pascual, Jacques Banchereau

**Affiliations:** 1 Baylor Institute for Immunology Research and Baylor Research Institute, 3434 Live Oak, Dallas, TX 75204, USA

## Abstract

Blood is the pipeline of the immune system. Assessing changes in transcript abundance in blood on a genome-wide scale affords a comprehensive view of the status of the immune system in health and disease. This review summarizes the work that has used this approach to identify therapeutic targets and biomarker signatures in the field of autoimmunity and infectious disease. Recent technological and methodological advances that will carry the blood transcriptome research field forward are also discussed.

## Profiling the human immune system

The immune system plays a central role not only in health maintenance but also in pathogenesis: excess immunity is associated, for instance, with auto-immune diseases (for example, multiple sclerosis, type 1 diabetes, psoriasis, lupus, rheumatoid arthritis), inflammation (sepsis, inflammatory bowel disease) and allergy, as well as cell and organ rejection; deficient immunity is, on the other hand, linked to cancer or susceptibility to infection.

**Figure 1:**
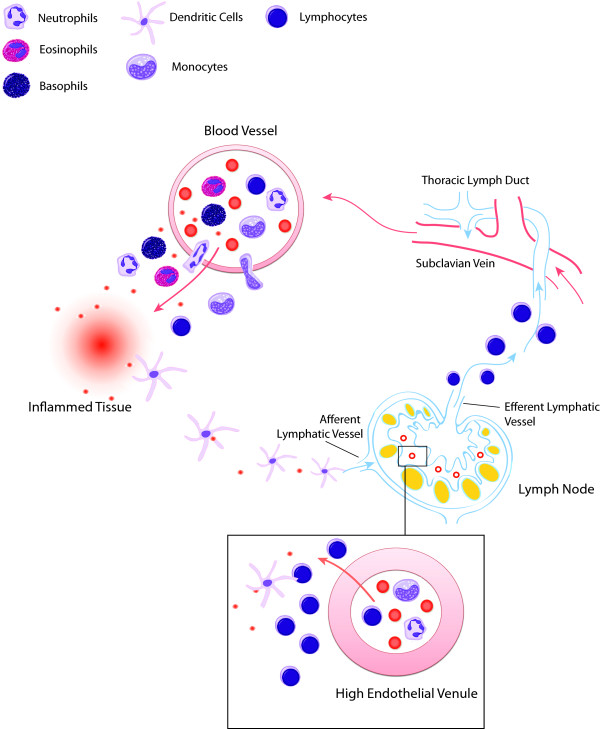
**Blood is the pipeline of the immune system**. Transcriptional profiling in the blood consists of measuring RNA abundance in circulating nucleated cells. Changes in transcript abundance can result from exposure to host or pathogen-derived immunogenic factors (for example, pathogen-derived molecular patterns activating specialized pattern recognition receptors expressed at the surface of leukocytes) and/or changes in relative cellular composition (for example, influx of immature neutrophils occurring in response to bacterial infection). The main blood leukocyte populations circulating in the blood are represented in this figure. Each cell type has a specialized function. Eosinophils, basophils and neutrophils are innate immune effectors playing a key role in defense against pathogens. T lymphocytes are the mediators of the adaptive cellular immune response. Antibody producing B lymphocytes (plasma cells) are key effectors of the humoral immune response. Monocytes, dendritic cells and B lymphocytes present antigens to T lymphocytes and play a central role in the development of the adaptive immune response. Blood leukocytes can be exposed in the circulation to factors released systemically from tissues where pathogenic processes take place. In addition, leukocytes will cross the endothelial barrier to reach local sites of inflammation. Dendritic cells exposed to inflammatory factors in tissues will be transported via the lymphatic system and reach lymph nodes via the afferent lymphatic vessels. These dendritic cells will encounter naïve T cells that are transported to the lymph node via high endothelial venules. 'Educated' T cells will then exit the lymph node via efferent lymph vessels that collect in the thoracic lymph duct, which in turn connects to the subclavian vein, at which point these T cells rejoin the blood circulation.

When investigating immune-mediated diseases in humans, restricted access to relevant tissue(s) for sampling, such as the brain in multiple sclerosis or the joints in rheumatoid arthritis, constitutes a major limitation. Cells of the immune system, however, become educated and implement their functions by recirculating between central and peripheral lymphoid organs as well as by migrating to and from sites of injury via the blood (Figure 1). As blood flows throughout the body, carrying naïve and educated immune cells from one site to another, it acts as a pipeline for the immune system. Indeed, it is the preferred route for immune cells to reach the lymph nodes where antigen-specific immune responses develop. After exiting these nodes through outgoing lymphatic vessels, the cells again reach the bloodstream to be transported to tissues throughout the body. Upon patrolling these tissues, they gradually drift back into the lymphatic system to re-enter the blood and begin the cycle all over again. The complex patterns of recirculation depend on the state of cell activation, the adhesion molecules expressed by immune and endothelial cells, and the presence of chemotactic molecules that selectively attract particular populations of blood cells. Circulating immune cells are, in addition, exposed to factors that are released systemically.

**Figure 2:**
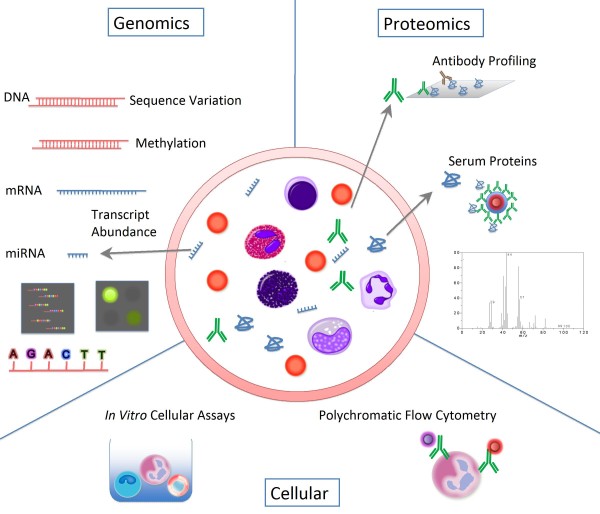
**The immune profiling armamentarium**. The number of high-throughput molecular and cellular profiling tools that can be used to profile the human immune system is increasing rapidly. Proteomic assays are used to determine antibody specificity or measure changes in serum levels of cytokines or chemokines using multiplex assays. Cellular profiling assays are used to phenotype immune cells based on intracellular or extracellular markers using polychromatic flow cytometry. *In vitro *cellular assays can measure innate or antigen-specific responsiveness in cells exposed to immunogenic factors. Genomic approaches consist of measuring abundance of cellular RNA and also microRNAs that are present in cells or in the serum. Other genomic approaches consist of determining gene sequence and function (for example, genome-wide association studies, RNA interference screens, exome sequencing).

A wide range of molecular and cellular profiling assays is currently available for the study of the human immune system (Figure 2). The level of sophistication of instruments such as polychromatic flow cytometers, one of the immunologist's favorite tools, has increased over the past few years. Major technological breakthroughs have also occurred in the fields of genomics and proteomics, thus creating today a unique opportunity for the study of human beings in health and disease where inherent heterogeneity dictates that large collections of samples be analyzed. Among the high-throughput molecular profiling technologies available today, genomic approaches are the most scalable, have the most breadth and robustness, and therefore are best suited for the study of human populations.

The human genome can be investigated from two different angles that consist of either determining its make up or measuring its output. Sequence variation can be detected using, for instance, single nucleotide polymorphism (SNP) chips, which permit the identification of common polymorphisms or rare mutations associated with diseases. Hundreds of thousands of SNPs can be typed using these platforms, yielding a genome-wide, hypothesis-free scan of genetic associations for a given phenotype of interest. Many such genome-wide association studies (often referred to as GWAS) have been published in recent years, a number of them investigating the genetic underpinning of immune-related diseases [1]. Notably, such studies have been useful to pinpoint genes and pathways that may be involved in the pathogenesis of autoimmune diseases [2]. Associations between common genetic variants and resistance to infection have also been reported [3,4]. However, parameters measured by this approach are determined by heredity and will not change throughout the life of an individual. This is in contrast to transcript abundance, which is the parameter measured by the second genome-wide profiling approach. Transcriptional activity is largely dependent on environmental factors and, as a result, RNA abundance will change dynamically over time. For instance, sets of transcripts may be induced in response to an infectious challenge and return to baseline levels following pathogen clearance. Dynamic changes in the cellular make up of a tissue will also effect changes in transcript abundance that will be measured on a genome-wide scale.

Transcriptional profiles have been obtained from many human tissues -including, for instance, the skin [5,6], muscle [7], liver [8,9], kidney [10,11] or brain [12] - but the status of the immune system can be best monitored by profiling transcript abundance in blood. Indeed, profiling transcript abundance in blood provides a 'snap shot' of the complex immune networks that operate throughout the entire body. However, while this has proven to be a valid approach to finding clues about pathogenesis as well as to identifying potential biomarkers [13-16], a number of challenges and limitations exist. Data interpretation is one of them. Firstly, the volume of data generated from such studies can be overwhelming, and it is necessary to integrate information from a multitude of sources (study design, quality control data, sample information, and importantly clinical information) in order for the results to be interpretable. Secondly, the changes in transcript abundance observed in complex tissues such as blood can be caused not only by regulation of gene transcriptional activity but also by relative changes in abundance of cell populations expressing transcripts at constant levels. Thirdly, in addition to pathogenic processes, a number of factors may affect blood transcript abundance and confound the analysis. Medications and co-morbidities are two such factors that often restrict patient selection and complicate data interpretation. This review will discuss some of the strategies recently developed that will address some of these limitations.

## Transcriptome profiling: a technology primer

**Figure 3:**
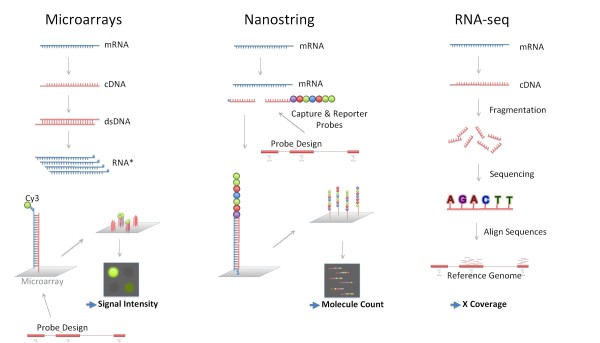
**RNA profiling technologies**. Several technology platforms are available for measuring RNA abundance on large scales. Microarray technologies rely on dense arrays of oligonucleotide probes used to capture complementary sequences present in biological samples at various concentrations. Following extraction, RNA is used as a template and amplified in a labeling reaction. The labeled material captured by the microarray is imaged and relative abundance determined based on the strength of the signal produced by the fluorochromes that serve as reporters in this assay. The Nanostring technology measures RNA abundance at the single molecule level. RNA serves as starting material for this assay, which does not involve the use of enzymes for amplification or labeling. Capture and reporter probes form complexes in solution with RNA molecules. These complexes are captured on a solid surface and imaged. Molecule counts are generated based on the number of reporter probes detected on the image. The reporter consists of a string of seven fluorochromes, with four different colors available to fill each position. Up to 500 different transcripts can be detected in a single reaction on this platform. For RNA sequencing (RNA-seq) the starting RNA population must first be converted into a library of cDNA fragments. High throughput sequencing of such fragments yields short sequences or reads that are typically 30 to 400 bp in length. For a given sample tens of millions of such sequences will then be uniquely mapped against a reference genome. The density of coverage for a given gene determines its relative level of expression. Similarities and differences between these technology platforms should be noted. For instance, microarrays and Nanostring technologies rely on oligonucleotide probes to capture complementary target sequences. Nanostring and RNA-seq technologies measure abundance at the single molecule level, with results expressed as molecule counts and sequence coverage, respectively. Microarray and RNA-seq technologies require extensive sample processing, which include amplification steps. dsDNA, double-stranded DNA.

Real-time PCR technology is currently considered the gold standard for the analysis of gene expression. However, it can be used to measure abundance of only a limited number of transcripts. Introduced over 10 years ago, DNA microarrays are now in routine use and can measure transcript abundance on a genome-wide scale. This technology relies on dense arrays of oligonucleotide probes that will capture complementary sequences present in biological samples at various concentrations. The probes can be deposited on a solid surface (printed microarrays), synthesized *in situ *(Affymetrix GeneChips), or bound to glass beads lodged into wells etched in the surface of a glass slide (Illumina BeadArrays). The labeled material captured by the microarray is imaged and relative abundance determined based on the strength of the signal produced by each oligonucleotide feature. It should be noted that, while they provide a means to survey transcript abundance on a genome-wide scale, the sensitivity of microarray assays is low compared to other approaches such as real-time PCR. A microarray is not a fully quantitative assay and changes in transcript abundance must be measured in reference to control samples that need to be included in each study. However, some of these limitations may be lifted by methods relying on high-throughput sequencing for the genome-wide measurement of RNA abundance [17]. Building on the legacy of the SAGE (serial analysis of gene expression) technology introduced in the 1990s, RNA sequencing (RNA-seq) [18] uses either total or fractionated RNA, for example poly(A)+, as a starting point. This material is converted to a library of cDNA fragments. High throughput sequencing of such fragments yields short sequences or reads that are typically 30 to 400 bp in length, depending on the technology platform used. For a given sample, tens of millions of such sequences will then be uniquely mapped against a reference genome. The higher the level of expression of a given gene, the higher the number of reads that will be aligned against it (Figure 3). Thus, this approach does not rely on probe design and provides several types of information, including not only transcript abundance but also transcriptome structure (splice variants), profiles of non-coding RNA species, and genetic polymorphisms. RNA-seq is expected to become sufficiently cost-effective and practical that it will eventually supersede microarray technologies.

Other technologies should be considered for the profiling of focused sets of genes. Nanostring technology can, for instance, detect the abundance of up to 500 transcripts with high sensitivity [19]. The approach is 'digital' since it counts individual RNA molecules using strings of fluorochromes as reporters to identify the different RNA species. Other technology platforms developed by, among others, Luminex, High Throughput Genomics or Fluidigm round up the offering for 'sub-genome' transcript profiling.

## Profiling autoimmune diseases

The field of autoimmunity has proven a fertile ground for blood transcriptional studies. Alterations in transcript abundance in the blood of patients reflect the sustained response against self-antigens and, more generally, uncontrolled inflammatory processes. Such diseases often present with recurring-remitting patterns of activity, with episodes of flaring that may be reflected by fluctuations in transcript abundance. The work has initially focused on diseases with clear systemic involvement such as systemic lupus erythematosus (SLE) [20,21]. Multiple cell types and soluble mediators, including IL10 [22,23] and IFNγ [24-26], have been proposed to be at the center of lupus pathogenesis. While some scattered evidence indicated the potential role of type I interferon in lupus, several observations did not support the hypothesis: first, not every SLE patient has detectable serum type I IFN levels [27]; second, dysregulation of type-I IFN production is not found in most murine SLE-models [28]; and third, genetic linkage and association studies had not identified candidate lupus susceptibility genes within the IFN pathway [29]. However, in one of our earliest microarray studies we demonstrated that all but one of the pediatric patients exhibited upregulation of IFN-inducible genes, and the only patient lacking this signature had been in remission for over 2 years [20]. In addition, it was found that treating SLE patients with high dose IV steroids, which are used to control disease flares, results in the silencing of the IFN signature. A surprise from these initial studies was the absence of type I IFN gene transcripts in the face of an abundance of IFN-inducible ones in the blood cells of SLE patients. A likely explanation is that the cells producing type I IFN, and therefore transcribing these genes, migrate to sites of injury. Altogether, results from microarray studies played a key role in convincing the community of the potential importance of type I IFN in SLE pathogenesis [15,30-34]. A phase Ia trial to evaluate the safety, pharmacokinetics, and immunogenicity of anti-IFN monoclonal antibody (mAb) therapy in adult SLE patients was recently conducted [35]. The antibody elicited a specific and dose-dependent inhibition of overexpression of type I IFN-inducible genes in both whole blood and skin lesions from SLE patients, at both the transcript and protein levels. As expected, overexpression of BLyS/BAFF, a type I IFN-inducible gene, also decreased with treatment. Thus, this first trial supports the proposed central role of type I IFN in human SLE.

Systemic onset juvenile arthritis (SoJIA) is another disease with systemic involvement that greatly benefited from the study of blood transcriptional profiles with the development of both therapeutic and diagnostic modalities [14,16,36,37]. Diseases with specific organ involvement have also been the subject of significant, yet not always extensive, blood profiling efforts. Blood signatures have, for instance, been obtained from patients with multiple sclerosis [38,39]. Given the inaccessibility of the brain, blood constitutes a particularly attractive source of surrogate molecular markers for this disease. These efforts have yielded a systemic signature and identified potential predictive markers of clinical relapse and response to treatment [40-42]. Transcriptional signatures have also been generated in the context of dermatologic diseases. In this case, the target organ being readily accessible, efforts have been focusing on profiling transcript abundance in skin tissues [43,44]. However, systemic involvement has been recognized in recent years to be an important component of autoimmune skin diseases and unique blood transcriptional profiles have also been identified in patients with, for example, psoriasis [45-47].

Blood transcriptional profiles have been generated in the context of many other autoimmune diseases. Indeed, the range of autoimmune/autoinflammatory diseases that have been investigated encompasses SLE [20,21,48,49], juvenile idiopathic arthritis [16,50-53], multiple sclerosis [54,55], rheumatoid arthritis [56-59], Sjogren's syndrome [60], diabetes [61,62], inflammatory bowel disease [63], psoriasis and psoriatic arthritis [45,47], inflammatory myopathies [64,65], scleroderma [66,67], vasculitis [68] and anti-phospholipid syndrome [69]. The body of work produced that focuses on blood transcript profiling in the context of autoimmune diseases has been covered at length in a recent review [70].

## Profiling infectious diseases

Global changes in transcript abundance have also been measured in the blood of patients with infectious diseases. In this context, alterations of blood transcriptional profiles are a reflection of the immunological response mounted by the host against pathogens. This response is initiated by specialized receptors expressed at the surface of host cells recognizing pathogen-associated molecular patterns [71]. Different classes of pathogens signal through different combinations of receptors, eliciting in turn different types of immune responses [72]. This translates experimentally into distinct transcriptional programs being induced upon exposure of immune cells *in vitro *to distinct classes of infectious agents [73-75]. Similarly, patterns of transcript abundance measured in the blood of patients with infections caused by different etiological agents were found to be distinct [13].

Predictably, dramatic changes were observed in the blood of patients with systemic infections (for example, sepsis) [76,77]. However, profound alterations in patterns of transcript abundance were also found in patients with localized infections (for example, upper respiratory tract infection, urinary tract infections, pulmonary tuberculosis, skin abscesses) [13,16,78]. Measuring changes in host transcriptional profiles may therefore prove of diagnostic value even in situations where the causative pathogenic agent is not present in the test sample. Importantly, it may also help ascertain the severity of the infection and monitor its course.

Infections often present as acute clinical events; thus, it is important to capture dynamic changes in transcript abundance that occur during the course of the infection from the time of initial exposure. Blood signatures have been described in the context of acute infections caused by a wide range of pathogenic parasites, viruses and bacteria, including *Plasmodium *[79,80], respiratory viruses (influenza, rhinovirus, respiratory syncytial virus) [13,81-84], dengue virus [85,86], and adenovirus [82], as well as *Salmonella *[87], *Mycobacterium tuberculosis *[78], *Staphylococcus aureus *[88], *Burkholderia pseudomallei *[76] and the general context of bacterial sepsis [77,89-91]. Some of those pathogens will persist and establish chronic infections (for example, human immunodeficiency virus and *Plasmodium*) that may lead to a state of latency (for example, tuberculosis), and transcript profiling may be used in those situations as a surveillance tool for monitoring disease progression or reactivation.

Blood profiling of infectious diseases remains limited in scale. In particular, additional studies will be necessary to ascertain dynamic changes occurring over time.

## Profiling other diseases

In addition to autoimmune and infectious diseases, blood transcript profiling studies have been carried out in the cancer research field. While hematological malignancies have led the way (reviewed in [92]), blood profiles have also been obtained more recently from patients with solid organ tumors [93]. Notably, these signatures can reflect not only the immunological or physiological changes effected by cancers but also the presence of rare tumor cells in the circulation [94-96].

Blood signatures have also been obtained from solid organ transplant recipients in the context of both tolerance [97-99] and graft rejection [10,100,101]. While such signatures can also be detected in biopsy material [102-104], blood offers the distinct advantage of being accessible for safely monitoring molecular changes on a routine basis.

Some work has also been done in the context of cardiovascular diseases where inflammation is known to play an important role. Hence, profiles have been identified in a wide range of conditions, including stroke, chronic heart failure or acute coronary syndrome [105-108].

The body of published work is too large to be cited in this review - and it is likely to be only the tip of the iceberg, with a lot more unpublished data scattered throughout public and private repositories. Other efforts have yielded, for instance, blood transcriptional signatures in patients with neurodegenerative diseases [109-111], and those associated with disease exacerbation or responsiveness to glucocorticoids in patients with asthma [112,113], and with responses to environmental exposure [114-116], exercise [117,118] or even laughter [119]. Unfortunately, too many published studies are underpowered and sometimes lack even the most rudimentary validation steps. All too often primary data are not available for reanalysis either, reflecting a lack of enforcement of editorial policies, or the absence thereof in some journals. Hence, one of the main challenges for this field is to move beyond the proof of principle stage and consolidate the wealth of data being generated.

Collectively, studies published thus far demonstrate that alterations in transcript abundance can be detected on a genome-wide scale in the blood of patients with a wide range of diseases. This statement is far from trivial given the skepticism that initially met studies investigating the blood transcriptome of patients. We have also learned that: 1) multiple diseases can share components of the blood transcriptional profile - for instance, the case for inflammation or interferon signatures; 2) while no single element of the profile may be specific to any given disease it is the combination of those elements that makes a signature unique; and finally, 3) the work accomplished to date highlights the importance of carrying out analyses aiming at directly comparing transcriptional profiles across diseases. Indeed, much can be learned, for instance, about autoimmunity from studying responses to infection, and vice versa. Furthermore, such efforts may eventually lead us closer to a molecular classification of diseases. First, however, technological and methodological advances are necessary for the blood transcriptome research field to move beyond the proof of principle stage.

## Moving forward

Recent progress in blood transcriptome research has been possible thanks to the development of robust sample collection techniques and the introduction of high throughput gene expression microarray platforms. Such advances have been necessary but the margin for progression in the field is still very significant. We describe here some of the current hurdles and discuss potential solutions for overcoming them.

### Data management

**Figure 4:**
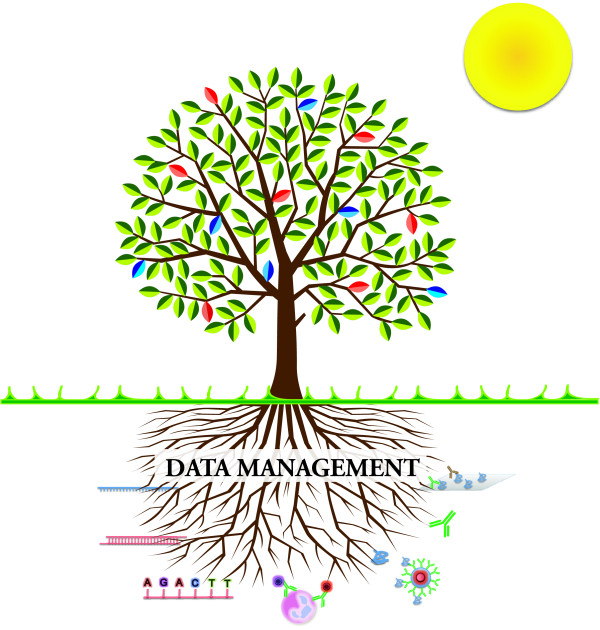
**Data management is key to progress**. Extensive cellular and molecular profiling of human subjects generates vast amounts of disparate data. Effective data management and integration solutions are essential to the preservation of this information in an interpretable form. Thus, data management efforts occurring 'behind the scenes' have an essential role to play in realizing the full potential of high throughput profiling approaches in human subjects.

For years the scale of blood transcriptional studies has been constrained by the cost of the technology. With the price tag on a commercial whole genome microarray below the $100 US mark, this is not the case anymore. Thus, data management has now become the first essential step to making large scale molecular profiling a viable proposition. Beyond storing the output of microarray instruments, data management must capture and organize information that is essential for the interpretation of the results (Figure 4). This includes sample information, data quality metrics, clinical information collected at the time of sampling, details about the experimental design, and materials and methods. Capturing such information ensures that the large volumes of data generated, which are often not published immediately, will remain exploitable for years to come. This point has become critical given the fact that results from genome-wide profiling studies can never be exploited to their fullest extent and possess considerable cumulative value when re-analyzed collectively. Notably, the results generated by other cellular and molecular profiling platforms will also need to be integrated in order to complete the picture. Therefore, implementing effective data management solutions and practices is essential to sustain the necessary increase in the scale of blood transcriptional studies (Figure 3) [120]. Unfortunately, implementing data management solutions in the laboratory is often an expensive proposition, requiring customization of off-the-shelf products or development of custom software adapted to handle specific workflows. Managing data also takes time and requires dedicated personnel. Thus, while the need is widely perceived, the commitment and steps necessary to implement effective data management solutions and practices are rarely adopted.

### Data mining

**Figure 5:**
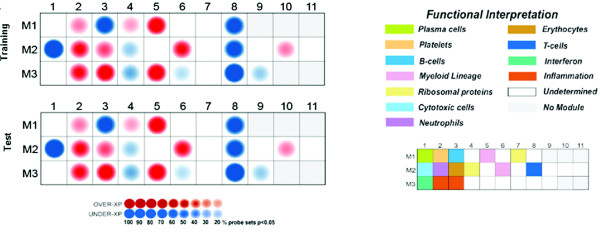
**Blood transcriptional fingerprints of patients with *Staphylococcus aureus *infection**. Relative changes in transcript abundance in the blood of patients with *S. aureus *infection compared to that of healthy controls are recorded for a set of 28 transcriptional modules. Colored spots represent relative increase (red) or decrease (blue) in transcript abundance (*P *< 0.05, Mann Whitney) within a module. The legend shows functional interpretation for this set of modules. Fingerprints have been generated for two independent cohorts of subjects (divided into a training set used in the discovery phase, n = 30, and an independent test set used in the validation phase, n = 32).

A myriad of approaches have been developed for the analysis of genome-wide transcriptional profiling data [121-124]. However, there is no silver bullet when it comes to microarray data analysis. The challenges encountered are several fold: 1) dimensionality, or how to cope with the fact that the number of parameters measured exceeds by several orders of magnitude the number of conditions included in most experiments; 2) noise - a direct consequence of the first point is that results from microarray analyses are particularly permissive to noise (false discovery); 3) 'seeing' the data - data visualization is critical as it helps promote insight and supports data interpretation; 4) biological context - it is important to keep the biology in sight at all times. Indeed, while it is easy to become absorbed by the data, it is essential to use biological knowledge when designing analysis strategies. Finally, there is hardly a one-size-fits-all approach to microarray data analysis and what works in one situation may not be universally applicable. Indeed, the most common response from experts when questioned on the best way to analyze a given dataset is that 'it depends...': it depends, for instance, on the extent of the differences being observed or on the variability inherent to a given disease or study population; it depends on what questions are being asked; or it can depend on whether follow-up confirmatory experiments are planned. In Table 1 we provide a data mining primer that explains the basic steps involved in microarray data analysis and the considerations that arise [125-129]. *Ad hoc *data mining approaches can be developed to meet specific needs. For instance, we have developed a data mining strategy for the specific purpose of analyzing blood transcriptional profiles [15]. This approach simply consists of *a priori *grouping of sets of genes with similar transcriptional patterns. This is repeated for several different datasets and subsequently, when comparing the cluster membership of all the genes across those datasets, the genes with similar membership are grouped together to form what we have termed a transcriptional module. Structuring the data permits focusing downstream statistical testing on these sets of transcripts that form coherent transcriptional and functional modular units. This is in contrast with more traditional approaches that rely on iterative statistical testing for thousands of individual transcripts that are treated as independent variables. The modular transcriptional framework that we have developed reduces the number of variables by collapsing sets of coordinately expressed genes into a new entity, the module. Reducing data dimensionality as such can: 1) facilitate functional interpretation; 2) enable comparative analyses across multiple datasets and diseases; 3) minimize noise and improve robustness of biomarker signatures; and 4) yield multivariate metrics that can be used at the bedside [15]. Data visualization is also of critical importance for the interpretation of large-scale datasets. We have devised a straightforward visualization scheme for mapping global transcriptional changes for individual diseases on a modular basis (Figure 5).Briefly, differences in expression levels between study groups are displayed for each module on a grid. Each position on the grid is assigned to a given module; a red spot indicates an increase and a blue spot a decrease in transcript abundance. The spot intensity is determined by the proportion of transcripts reaching significance for a given module. *A posteriori*, biological interpretation has linked several modules to immune cells or pathways (see legend of Figure 5). Hence, in the example provided in Figure 5, patients with *S. aureus *infection demonstrate significant over-expression of genes in modules related to innate immunity, including myeloid (M1.5, M2.6), neutrophil (M2.2), and inflammation (M3.2, M3.3) modules, and under-expression of genes regulating adaptive immunity such as those in B cell (M1.3), cytotoxic cell (M2.1), and T-cell-specific (M2.8) modules. It should also be noted that no changes were observed for other modules, such as module M3.1, which includes interferon-inducible genes, abundance of which would be increased in the context of a viral infection.

**Table 1:** A data mining primer: basic steps used for analysing microarray data

Here we provide basic analysis steps and important considerations for microarray data analysis:
- Per-chip normalization: This step controls for array-wide variations in intensity across multiple samples that form a given dataset. Arrays, as with all fluorescence based assays, are subject to signal variation for a variety of reasons, including the efficiency of the labeling and hybridization reactions and possibly other, less well defined variables, such as reagent quality and sample handling. To control for this, samples are normalized by first subtracting background and then employing a normalization algorithm to rescale the difference in overall intensity to a fixed intensity level for all samples across multiple arrays.
- Data filtering: Typically more than half of the oligonucleotide probes present on a microarray do not detect a signal for any of the samples in a given analysis. Thus, a detection filter is applied to exclude these transcripts from the original dataset. This step avoids the introduction of unnecessary noise in downstream analyses.
- Unsupervised analysis: The aim of this analysis is to group samples on the basis of their molecular profiles without *a priori *knowledge of their phenotypic classification. The first step, which functions as a second detection filter, consists of selecting transcripts that are expressed in the dataset and display some degree of variability, which will facilitate sample clustering. For instance, this filter could select transcripts with expression levels that deviate by at least two-fold from the median intensity calculated across all samples. Importantly, this additional filter is applied independently of any knowledge of sample grouping or phenotype, which makes this type of analysis 'unsupervised'. Next, pattern discovery algorithms are often applied to identify 'molecular phenotypes' or trends in the data.
- Clustering: Clustering is commonly used for the discovery of expression patterns in large datasets. Hierarchical clustering is an iterative agglomerative clustering method that can be used to produce gene trees and condition trees. Condition tree clustering groups samples based on the similarity of their expression profiles across a specified gene list. Other commonly employed clustering algorithms include k-means clustering and self-organizing maps.
- Class comparison: Such analyses identify genes that are differentially expressed among study groups ('classes') and/or time points. The methods for analysis are chosen based on the study design. For studies with independent observations and two or more groups, *t*-tests, ANOVA, Mann-Whitney U tests, or Kruskal-Wallis tests are used. Linear mixed model analyses are chosen for longitudinal studies.
- Multiple testing correction: Multiple testing correction (MTC) methods provide a means to mitigate the level of noise in sets of transcripts identified by class comparison (in order to lower permissiveness of false positives). While it reduces noise, MTC promotes a higher false negative rate as a result of dampening the signal. The methods available are characterized by varying degrees of stringency, and therefore they produce gene lists with different levels of robustness.
• Bonferroni correction is the most stringent method used to control the familywise error rate (probability of making one or more type I errors) and can drastically reduce false positive rates. Conversely, it increases the probability of having false negatives.
• Benjamini and Hochberg false discovery rate [125] is a less stringent MTC method and provides a good balance between discovery of statistically significant genes while limiting false positives. By using this procedure with a value of 0.01, 1% of the statistically significant transcripts might be identified as significant by chance alone (false positives).
- Class prediction: Class prediction analyses assess the ability of gene expression data to correctly classify a study subject or sample. K-nearest neighbors is a commonly used technique for this task. Other available class prediction procedures include, but are not limited to, discriminant analysis, general linear model selection, logistic regression, distance scoring, partial least squares, partition trees, and radial basis machine.
- Sample size: The number of samples necessary for the identification of a robust signature is variable. Indeed, sample size requirements will depend on the amplitude of the difference between, and the variability within, study groups.
A number of approaches have been devised for the calculation of sample size for microarray experiments, but to date little consensus exists [126-129]. Hence, best practices in the field consist of the utilization of independent sets of samples for the purpose of validating candidate signatures. Thus, the robustness of the signature identified will rely on a statistically significant association between the predicted and true phenotypic class in the first and the second test sets.

### Beyond mRNA: profiling microRNAs

MicroRNA (miRNA) control has emerged as a critical regulatory circuit of the immune system. Measuring changes in miRNA abundance in the blood of human subjects in health and disease is therefore a promising new field of investigation. These short non-coding single-stranded RNAs about 22 nucleotides in length have been found to play essential regulatory roles [130-132]. These molecules exhibit highly specific, regulated patterns of expression and control protein expression by translational repression, mRNA cleavage, or promotion of mRNA decay. Interestingly, thanks to their small size, miRNA molecules are stable and can be measured not only in blood cells but also in circulation in the serum [133]. They are thus not only potentially important contributors to immune function, but also potential sources of biomarkers.

### Deconvoluting blood transcriptional signatures

Blood transcriptome research will also benefit from conceptual advances that may help address shortcomings inherent to whole blood profiling.

First, blood is a complex tissue and changes in transcript abundance can be attributed to either transcriptional regulation or relative changes in composition of leukocyte populations. Two approaches exist for 'deconvoluting' these two phenomena. First, one can isolate and individually profile different cell populations present in the blood. This approach may also permit the identification of transcripts expressed at low levels or the detection of differences in expression that would otherwise be drowned in whole blood [134,135]. However, isolation methods may introduce technical bias, and require extensive sample processing. A second approach consists of deconvoluting whole blood transcriptional profiles '*in silico*'. This type of analysis attempts to deduce cellular composition or cell-specific levels of gene expression using statistical methodologies [136-141].

Finally, we must also keep in mind that the immune status of a human subject is not entirely reflected by its blood profile obtained at the steady state. Indeed, an individual's capacity to respond to innate as well as antigen-specific immune signals may also provide useful and complementary information.

In conclusion, blood transcript profiling has earned its place in the molecular and cellular profiling armamentarium used to study the human immune system. Changes in transcript abundance recapitulate the influence of genetic, epigenetic, cellular and environmental factors. Initially considered to belong to the 'cutting edge', this approach has become both robust and practical. As discussed in this review, it has become a mainstay for the study of immune function in patients with a wide range of diseases. Furthermore, recent studies have demonstrated the utility of blood transcriptome profiling for monitoring immune responses to drugs or vaccines [35,142,143]. Thus, blood transcript profiling is developing into a mainstream tool for the assessment of the status of the human immune system.
